# PD Koch Complementary Fractal UHF Antenna Based on AMC Metasurface

**DOI:** 10.3390/s26041398

**Published:** 2026-02-23

**Authors:** Haonan Zhang, Dapeng Han, Minghan Ke, Lihao Luo, Zhenhao Huang, Guozhi Zhang

**Affiliations:** Hubei Engineering Research Center for Safety Monitoring of New Energy and Power Grid Equipment, Hubei University of Technology, Wuhan 430068, China; 13035139268@163.com (H.Z.); 17622335969@163.com (D.H.); 13387560942@163.com (M.K.); 18102336261@163.com (L.L.); 13018053772@163.com (Z.H.)

**Keywords:** power equipment, partial discharge, ultra high frequency, Koch antenna, metasurface manipulation

## Abstract

To meet the high-sensitivity requirement of ultra-high-frequency (UHF) sensors for electromagnetic waves radiated by partial discharge (PD) in power equipment of substations, this paper proposes a Koch complementary fractal UHF antenna based on the artificial magnetic conductor (AMC) metasurface. First, based on the Iterated Function System (IFS), a finite element model of the UHF Koch fractal antenna is constructed via affine transformation. Then, leveraging the in-phase reflection characteristic of the metasurface, an AMC metasurface for gain enhancement of the Koch fractal antenna is designed, and a multi-dimensional parameter joint optimization method is adopted to obtain the optimal structural parameter set of the Koch fractal antenna loaded with the AMC metasurface. Finally, experimental tests and analyses are carried out on the Koch complementary fractal UHF antenna. The results show that the antenna loaded with the AMC metasurface achieves a better voltage standing wave ratio (VSWR) and improved gain in both low and high frequency bands: the average gain increases by 35.19% in the frequency range of 0.3 GHz to 1.5 GHz, and the peak gain reaches approximately 11.5 dB with an enhancement of 120%.

## 1. Introduction

As a core component of the power system, substations play an important role in transmission, distribution, and voltage transformation, as well as providing users with continuous, reliable, high-quality electricity. However, during the manufacturing, transportation, and maintenance of electrical equipment, insulation defects are inevitably generated. As the insulation materials of the equipment age, partial discharge (PD) becomes a major threat to the reliability and safety of high-voltage electrical equipment in substations [1,2].

During the partial discharge (PD) process, characteristic signals such as acoustic, magnetic, optical, electrical, and chemical phenomena are generated. The corresponding typical PD detection methods mainly include the pulse current method [3], ultrasonic detection method [4], ultra-high-frequency (UHF) method [5], optical detection method [6], and chemical detection method [7,8]. Among these methods, the UHF method stands out among various monitoring methods due to its high sensitivity and strong anti-interference capability [9].

High-sensitivity UHF antenna sensors are key to effectively perceiving PD radiation electromagnetic pulse signals. Typical UHF antenna sensors include helical antennas [10], microstrip monopole antennas [11], loop antennas [12], and Koch fractal antennas [13]. Among them, Koch fractal antennas have self-similarity, giving them wide bandwidth and compact features, leading to widespread application [14]. Reference [15] designed a Koch hexagonal fractal ring-slot array antenna, which uses a parallel power divider network for feeding, achieving high directivity and high gain, but its bandwidth is narrow and the standing wave ratio is poor; Reference [16] designed a new ultra-wideband fractal antenna, combining fractal technology with coplanar waveguide technology to achieve ultra-wideband characteristics, but its overall gain is poor and decreases at high frequencies; Reference [17] designed a substrate-integrated waveguide slot antenna with multiband characteristics, using substrate-integrated waveguide for impedance matching, but its sensitivity is low and gain is poor. Reference [18] designed a slotted dual-band wideband Koch fractal antenna, increasing bandwidth by adding a ‘U’-shaped slot to the main radiating element, but it has a poor standing wave ratio and low gain at low frequencies; Reference [19] designed an ultra-wideband Koch fractal patch antenna, enhancing bandwidth by adding rectangular slots and Koch snowflake fractal notches on the patch, but its gain across the full frequency band is low and sensitivity is poor; Reference [20] designed a miniaturized Koch-Minkowski hybrid fractal antenna, broadening the bandwidth by combining Koch and Minkowski fractals, but its overall gain is low and sensitivity is poor; Reference [21] designed a strip-type reconfigurable fractal antenna with partial ground modification, achieving miniaturization and broadband functionality through a star-shaped fractal geometry, but its gain is poor and sensitivity is low. Reference [22] designed a second-order pre-fractal Koch-type linear dipole antenna by adjusting the arm length, scaling ratio, and central vertex position. Using a Wi-Fi band antenna miniaturization algorithm, the optimal size was obtained, but the sensitivity was relatively poor. Reference [23] designs and optimizes a batwing-structured microstrip antenna to achieve ultra-wideband and miniaturization, suitable for PD signal reception in devices such as GIS, but its gain is relatively poor. Reference [24] designed a miniaturized triple ultra-wideband Sierpinski hexagonal fractal antenna, which can cover multiple wireless communication frequency bands, but the antenna lacks sensitivity. Reference [25] designed a miniaturized Koch fractal antenna, achieving wide impedance matching through fractal iteration and structural optimization, but the gain is relatively poor. Reference [26] designed a flexible Hilbert fractal antenna for GIS partial discharge UHF detection, featuring wide bandwidth, flexibility, and easy installation. It can effectively capture partial discharge signals but has relatively low sensitivity. Reference [27] selects substrate materials for built-in flexible UHF partial discharge through compatibility experiments with fault gases.

To address the problem of insufficient sensitivity of UHF fractal antennas for partial discharge (PD) detection in substation power equipment, this paper proposes a PD Koch complementary fractal ultra-high-frequency (UHF) antenna based on artificial magnetic conductor (AMC) metasurfaces. A finite element model of the UHF Koch fractal antenna is constructed via affine transformation. Then, leveraging the in-phase reflection characteristics of metasurfaces, an AMC metasurface structure for improving the gain of the Koch fractal antenna is designed. A multi-dimensional parameter joint optimization method is adopted to obtain the optimal set of structural parameters for the Koch fractal antenna loaded with the AMC metasurface. Finally, comparative experimental measurements and analyses of the Koch complementary fractal UHF antenna are conducted through electrostatic gun discharge experiments and insulation defect discharge experiments.

## 2. Modeling of the PD Koch Fractal Antenna

### 2.1. Design Principle of the Koch Fractal Antenna

**Figure 1:**
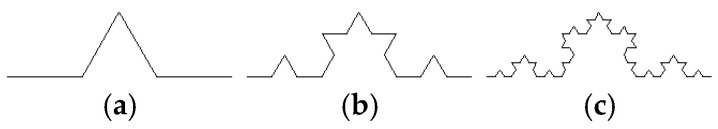
Fractal generation process of the Koch curve. (**a**) 1st-order fractal, (**b**) 2nd-order fractal, (**c**) 3rd-order fractal.

The Koch curve has strong space-filling property and self-similarity, thus attracting extensive attention in the design of multi-band and miniaturized antennas. The Koch curve is an irregular curve, whose basic principle is to trisect the initial straight line, rotate the middle segment counterclockwise by 60 degrees around its left endpoint and clockwise by 60 degrees around its right endpoint, and finally connect these newly generated straight lines end to end to form a higher-order fractal curve, as shown in Figure 1. Each iteration of the Koch curve increases its total length to 4/3 times the original length [13,14]. After n iterations, the total length $L_{n}$ of the curve is

(1) $$L_{n}=l_{0}\times {\left(4/3\right)}^{n}$$

where $l_{0}$ is the length of the initial straight line, and n is the fractal order.

Fractal dimension can be used to quantify the complexity and space-filling capacity of irregular and self-similar geometric bodies. The fractal dimension $D_{m}$ of the Koch curve is

(2) $$D_{m}=\log N_{m}/\log S_{m}$$

where $N_{m}$ is the number of generated units, and $S_{m}$ is the reciprocal of the scaling factor, with its value expressed as

(3) $$S_{m}=2\left(1+\cos\theta \right)$$

where θ = 60° is the rotation angle.

The construction method of the Koch complementary fractal curve is usually implemented by the Iterated Function System (IFS), which is a cluster of contractive affine transformations and can construct and describe a large class of fractal sets, including Koch structural curves. The Koch curve can be implemented by the following method:

(4) $$W\left(\begin{array}{c} x\\ y \end{array}\right)=\left(\begin{array}{cc} a & b\\ c & d \end{array}\right)\left(\begin{array}{c} x\\ y \end{array}\right)+\left(\begin{array}{c} e\\ f \end{array}\right)=A\left(\begin{array}{c} x\\ y \end{array}\right)+\left(\begin{array}{c} e\\ f \end{array}\right)$$

Here, *x* and *y* represent the endpoint coordinates of the segment line. The rotation angles and scaling factors *a*, *b*, *c*, and *d* controlling the segment line can be expressed by matrix *A*. The cosine values of the rotation angles and scaling factors are represented by *a* and *d*; the sine values of the rotation angles and scaling factors are represented by *b* and *c*, as shown in Formula (5). The offset constants *e* and *f* denote the displacement coefficients along the coordinate directions. For the Koch curve, the following relationships hold:

(5) $$A=\left(\begin{array}{cc} a & b\\ c & d \end{array}\right)=\left(\begin{array}{cc} r_{0}\cos\theta_{0} & -r_{1}\sin\theta_{1}\\ r_{0}\sin\theta_{0} & r_{1}\cos\theta_{1} \end{array}\right)$$

where $r_{0}$ = $r_{1}$ = 1/3 is the scaling factor, and $\theta_{0}$ = $\theta_{1}$ = 60° is the rotation angle. Therefore,

(6) $$W_{1}\left(\left.x,y\right)\right.=\left(\begin{array}{cc} 1/3 & 0\\ 0 & 1/3 \end{array}\right)\left(\begin{array}{c} x\\ y \end{array}\right)$$

(7) $$W_{2}\left(x,y\right)=\left(\begin{array}{cc} 1/6 & -\sqrt{3}/6\\ \sqrt{3}/6 & 1/6 \end{array}\right)\left(\begin{array}{c} x\\ y \end{array}\right)+\left(\begin{array}{c} 1/3\\ 0 \end{array}\right)$$

(8) $$W_{3}\left(x,y\right)=\left(\begin{array}{cc} 1/6 & \sqrt{3}/6\\ -\sqrt{3}/6 & 1/6 \end{array}\right)\left(\begin{array}{c} x\\ y \end{array}\right)+\left(\begin{array}{c} 1/2\\ \sqrt{3}/6 \end{array}\right)$$

(9) $$W_{4}\left(x,y\right)=\left(\begin{array}{cc} 1/3 & 0\\ 0 & 1/3 \end{array}\right)\left(\begin{array}{c} x\\ y \end{array}\right)+\left(\begin{array}{c} 2/3\\ 0 \end{array}\right)$$

The Koch curve can be obtained by combining $W_{1}\left(\left.x,y\right)\right.$, $W_{2}\left(x,y\right)$, $W_{3}\left(x,y\right)$ and $W_{4}\left(x,y\right)$.

### 2.2. Modeling and Simulation of the PD Koch Complementary Fractal Antenna

**Figure 2:**
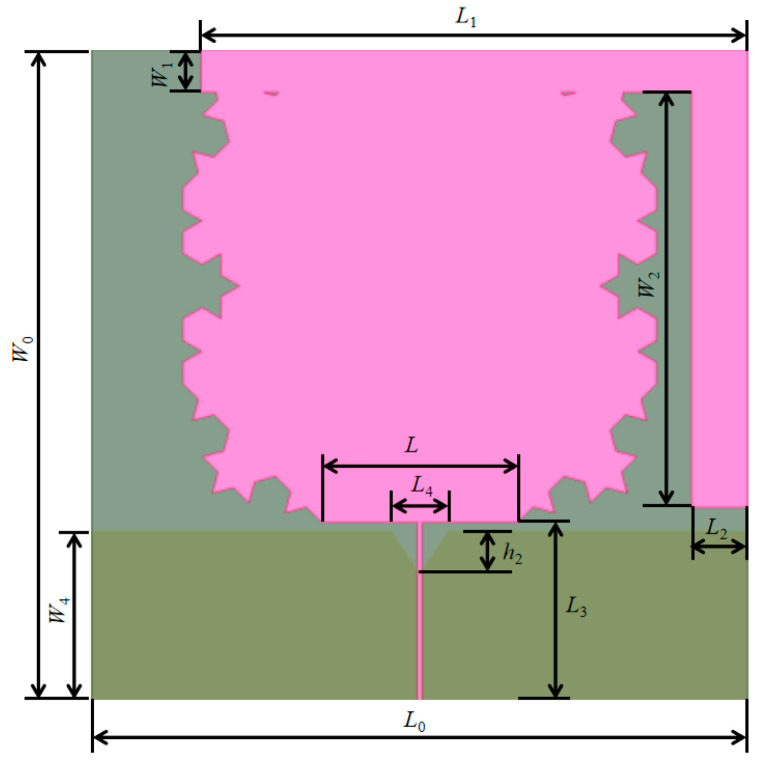
Koch fractal antenna modeling. The pink part is the Koch fractal antenna, the dark green part is the etched triangular ground plane, and the light green part is the dielectric substrate.

Given that the lowest frequency of the antenna is 300 MHz, to meet the design requirements for the electrical length of the antenna, an FR-4 dielectric substrate with length *a* and width *b,* both 180 mm, and height *h*_0_ at 1.6 mm, was selected. A regular octagon with side length *L* of 58.3 mm was subjected to a second-order fractal processing. The sides of the regular octagon were numbered in the clockwise rotation direction: sides 1 and 3 were processed with a second-order outward fractal, while side 2 was processed with a second-order inward fractal. Finally, the modeling of the entire antenna was completed through mirror operation, resulting in a complementary fractal structure. Above the antenna, an asymmetric stub 1 with length *L*_1_ of 150 mm and width *W*_1_ of 11.8 mm and an asymmetric stub 2 with length *L*_2_ of 15.372 mm and width *W*_2_ of 120 mm were loaded to increase the electrical length required for the low-frequency band. The antenna was fed by a 50-ohm microstrip line with a length *L*_3_ of 50 mm and a width *W*_3_ of 1.07 mm. On the back of the dielectric substrate, a ground plane with the same length as the substrate and a width *W*_4_ of 47.6 mm was adopted, and an equilateral triangular groove with a length *L*_4_ of 15.75 mm and a height *h*_2_ of 12 mm was etched in the middle to form a tapered feeding network. The radiation pattern is solved by using the air cavity of the Koch fractal antenna with a distance of λ_min_/4 length and the radiation boundary. The antenna model and antenna parameters are shown in Figure 2 and Table 1, respectively.

**Figure 3:**
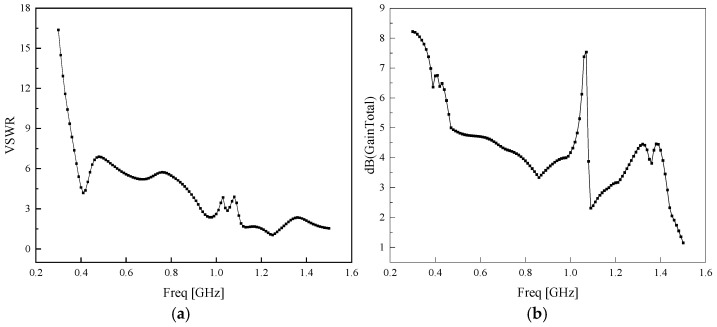
(**a**) Standing wave ratio of Koch fractal antenna; (**b**) gain of Koch fractal antenna.

After the modeling is completed in the electromagnetic simulation software HFSS 15.0, the standing wave ratio and gain characteristic parameters of the Koch fractal antenna obtained through simulation are shown in Figure 3, respectively.

As can be observed from Figure 3b, the Koch fractal antenna suffers from inferior gain performance across the mid- and high-frequency bands. To address this limitation and enhance the overall antenna performance, an artificial magnetic conductor (AMC) metasurface is integrated into the antenna structure in this work for gain improvement.

## 3. AMC Metasurface Regulation of PD Koch Fractal Antenna

### 3.1. Principle of Antenna Gain Enhancement via AMC Metasurfaces

**Figure 4:**
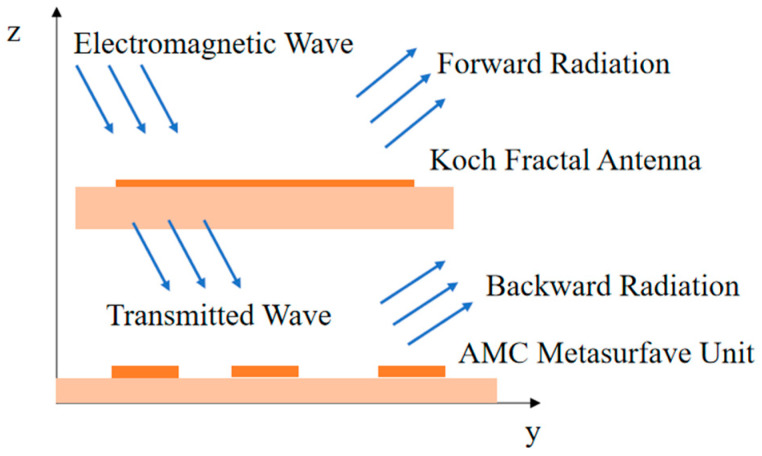
Schematic diagram of the AMC metasurface.

The performance enhancement of antennas using AMC metasurfaces has already been widely studied. Reference [28] employs a tunable AMC as the antenna ground plane, simultaneously achieving three major functions: bandwidth doubling, low profile, and out-of-band RCS reduction. Reference [29] combines AMC with PB phase-coded metasurfaces to achieve high gain at dual-frequency bands while also reducing RCS. Reference [30] first proposed the AMC metasurface, whose classic “mushroom” structure exhibits in-phase reflection within the bandgap, rather than the out-of-phase reflection characteristic of an ideal conductor. Reference [31] designed a low-profile, wideband, circularly polarized antenna assisted by a metasurface, achieving antenna gain enhancement, profile reduction, and wideband circular polarization performance with the help of an AMC structure. Reference [32] proposes a scattering matrix-based inverse design method for the rapid optimization of multi-port electromagnetic bandgap (EBG) waveguides and filters, allowing structural parameters to be directly derived from the desired electromagnetic response, thereby improving design efficiency and stability. Reference [33] designed a slot antenna based on an AMC metasurface loading, aiming to achieve a low radar cross section (RCS), effectively reducing the antenna’s RCS over a wide frequency band while maintaining good radiation performance. Reference [34] designed a low-profile circularly polarized antenna and enhanced its bandwidth and gain by loading a honeycomb-shaped AMC structure. This paper uses the AMC metasurface with the same reflection characteristic, which can reflect the backward radiation of the antenna and superimpose the forward radiation, so as to improve the gain of the antenna. When positioned beneath the antenna, the AMC metasurface reflects part of the incident electromagnetic waves (forward radiation) while transmitting the remainder. The transmitted waves undergo additional reflection (backward radiation) on the AMC metasurface. Due to their AR properties, the backward and forward radiations maintain phase coherence, enabling superposition that amplifies the forward radiation field. This process ultimately boosts the antenna’s gain [35,36]. The schematic is illustrated in Figure 4.

### 3.2. Modeling and Simulation of AMC Metasurface Units

**Figure 5:**
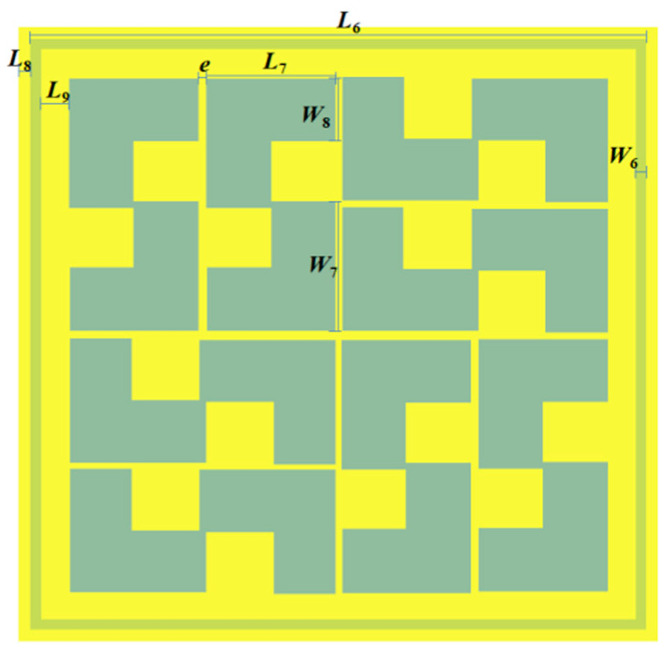
Model diagram of the AMC metasurface unit. The yellow part is the dielectric substrate, the dark green part is the S-type AMC metasurface unit, and the light green part is the square ring on the back.

Given the large size of the Koch fractal antenna, the AMC metasurface unit was designed to ensure that the electromagnetic waves incident on the metasurface unit are uniformly reflected back to the Koch fractal antenna. The AMC metasurface unit adopted FR-4 as the dielectric substrate with a length *L*_5_ = 184 mm, width *W*_5_ = 184 mm, height *h*_2_ = 1 mm and relative permittivity *ε*_r_ = 4.4. The front side of the FR-4 dielectric substrate featured a centrosymmetric pattern composed of 8 identical “S”-shaped patches, while the back side was a square metal ring. The square metal ring had a length of *L*_6_ = 53 mm and a width of *W*_6_ = 0.9 mm. Each “S”-shaped patch had a length of *L*_7_ = 11.1 mm and a width of *W*_7_ = 11.7 mm, and the width of each rectangular segment forming the “S”-shaped patch was *W*_8_ = 5.6 mm. The gap between two adjacent “S”-shaped patches was *e* = 0.6 mm, the distance from the square ring to the left edge of the dielectric substrate was *L*_8_ = 2 mm, the distance between the metasurface unit and the square ring was *L*_9_ = 2.5 mm, and the distance between adjacent metasurface units was *L*_10_ = 11 mm. The dielectric substrate was placed *h*_3_ = 30 mm below the antenna. The dimensional parameters and model of the AMC metasurface unit are presented in Table 2 and Figure 5, respectively.

**Figure 6:**
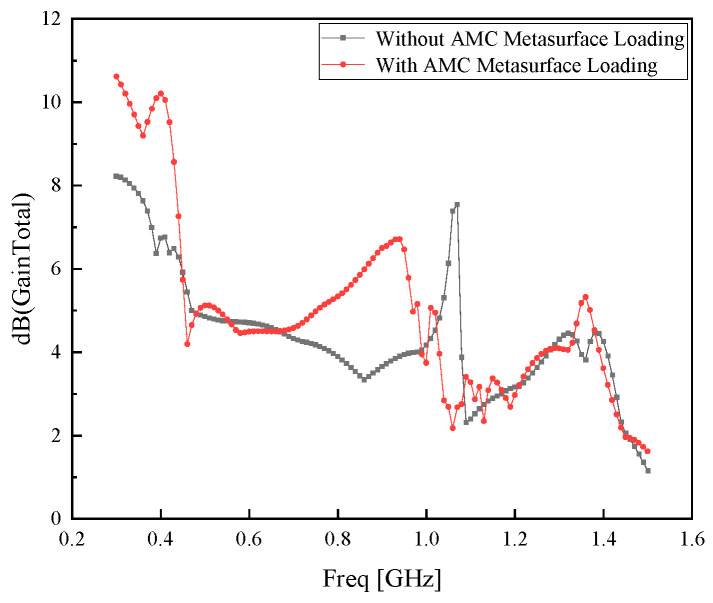
Gain comparison diagram of the antenna with and without AMC metasurface loading.

The preliminary comparison diagram of the gain of the Koch fractal antenna before and after loading the AMC metasurface, obtained via Ansoft HFSS simulation, is illustrated in Figure 6. As can be observed from the diagram, the gain of the antenna with the AMC metasurface is higher than that without the AMC metasurface, yet the overall improvement is relatively modest.

### 3.3. Optimization of Dimensional Parameters for AMC Metasurface Units

**Figure 7:**
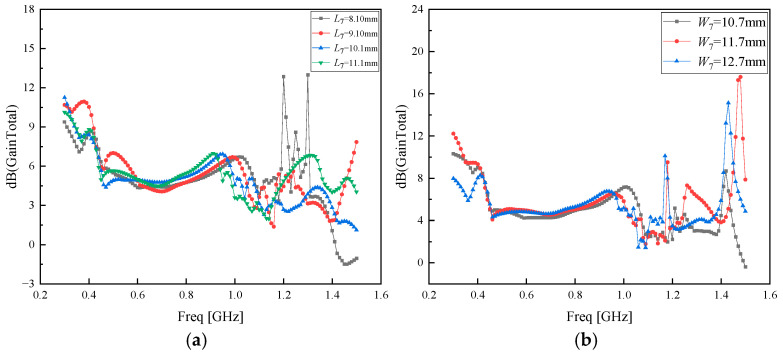
(**a**) Optimized parameter *L*_7_; (**b**) optimized parameter *W*_7_.

**Figure 8:**
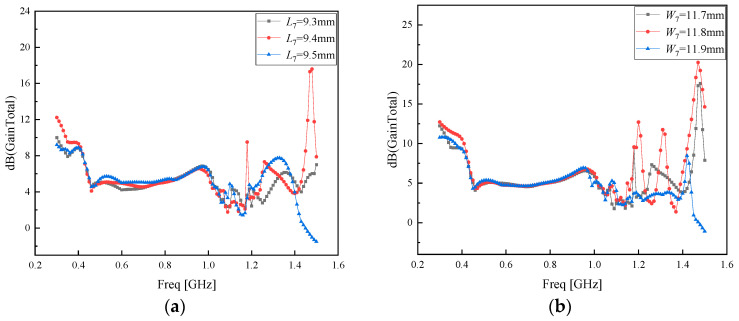
(**a**) Optimizing parameter *L*_7_ with a step size of 0.1 mm; (**b**) optimizing parameter *L*_7_ with a step size of 0.1 mm.

To further enhance the antenna gain of the PD Koch fractal antenna after metasurface modulation, the parameters of the metasurface units primarily affecting gain were optimized to achieve optimal gain. Reference [37] employs the multipole expansion and image method to efficiently and accurately solve the electromagnetic scattering from parallel cylinders above a planar conducting surface, replacing time-consuming full-wave numerical simulations; reference [38] simplified the simulation process by using electromagnetic simulation-driven parameterization and multi-objective collaborative optimization; reference [39] designed a metasurface based on Huygens’ principle and verified it using manual parameter scanning and full-wave simulation, demonstrating good rigor. In this paper, since the length *L*_7_ and width *W*_7_ of the “S”-shaped patch, along with the length *L*_6_ and width *W*_6_ of the square ring on the back of the dielectric plate, influence its effective area for receiving electromagnetic waves, the distance *L*_8_ between the square ring and the edge of the dielectric plate, the distance *L*_9_ between the S-shaped patch and the square ring edge, and the gap e between super-surface units all affect their distribution positions. The length *L*_11_ of the connecting bracket also influences gain. Therefore, a multi-parameter joint optimization method was employed. The specific parameters optimized are listed in Table 3, and the specific parameter optimization process is shown in Figure 7 and Figure 8, and Appendix A.

As can be observed from Figure 7, the AMC-regulated PD Koch fractal antenna achieves the optimal gain improvement effect when *L*_7_ = 9.10 mm and *W*_7_ = 11.7 mm. Considering that parameters *L*_7_ and *W*_7_ are related to the area of the metasurface unit, further optimization was conducted around *L*_7_ = 9.10 mm and *W*_7_ = 11.7 mm with a step size of 0.1 mm. The optimization results of *L*_7_ and *W*_7_ are presented in Figure 8a,b, respectively.

As can be observed from Figure 8, the AMC-regulated PD Koch fractal antenna achieves the optimal gain improvement effect when *L*_7_ = 9.4 mm and *W*_7_ = 11.8 mm. For the sake of conciseness and clarity, the optimization of the remaining parameters can be found in Appendix A.

**Figure 9:**
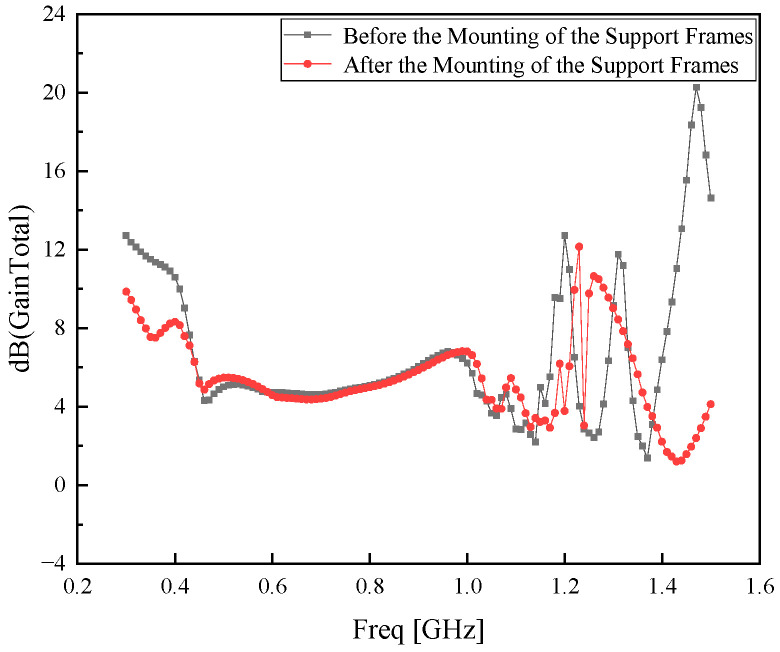
Gain comparison diagram with and without support frames.

The entire metasurface is connected to the Koch fractal antenna via four support frames made of FR-4 material, with an initial length of *L*_11_ = 20.0 mm. The gain comparison before and after the addition of the support frames is illustrated in Figure 9.

**Figure 10:**
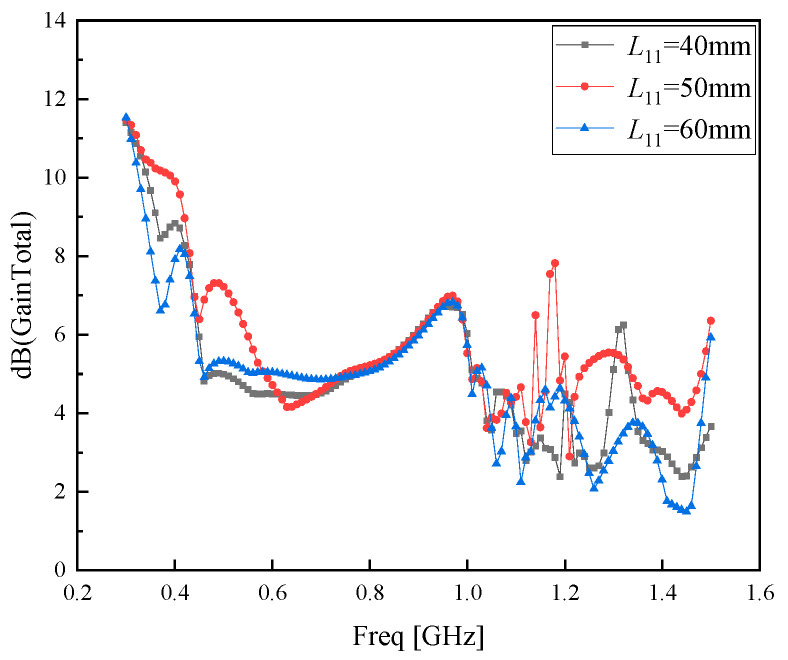
Optimized parameter *L*_11_.

As can be observed from Figure 9, the overall gain of the antenna deteriorates with the installation of the support frames. To minimize the impact of the support frames on the antenna gain as much as possible, the length *L*_11_ of the support frames is optimized based on the parameter values of *L*_7_, *W*_7_, *e*, *L*_9_, *W*_6_, *W*_8_, *L*_8_, *L*_10_, and *h*_3_. The optimization diagram of parameter *L*_11_ is illustrated in Figure 10.

As can be observed from Figure 10, the length of the support frames exerts the minimal impact on the gain when *L*_11_ = 50 mm. The comparison between the finally optimized parameter values and the original ones is presented in Table 4.

**Figure 11:**
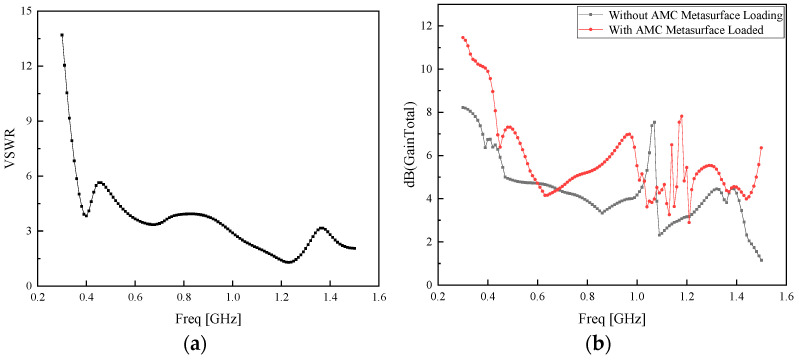
(**a**) Standing wave ratio of the Koch fractal antenna loaded with the optimized AMC metasurface; (**b**) Gain comparison with and without the optimized AMC metasurface.

The standing wave ratio of the Koch fractal antenna loaded with the optimized AMC metasurface is illustrated in Figure 11a. The gain comparison between the Koch fractal antenna without the AMC metasurface and that loaded with the optimized AMC metasurface is presented in Figure 11b.

As can be seen from Figure 11, the standing wave ratio of the antenna decreases in the low-frequency band after loading the AMC metasurface, and the gain is enhanced over almost the entire frequency band. The average gain is increased by 35.19%, and the peak gain reaches approximately 11.5 dB with an improvement of 120%, yielding a remarkable performance improvement.

**Figure 12:**
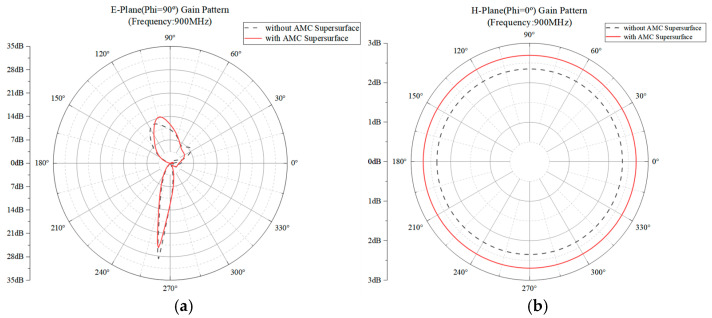
Comparison of two-dimensional E/H-plane radiation patterns at 900 MHz before and after AMC Supersurface loading. (**a**) Comparison of E-plane radiation before and after loading at 900 MHz. (**b**) Comparison of H-plane radiation before and after loading at 900 MHz.

The E/H plane radiation before and after loading the AMC super table at the center frequency of 600 MHz is shown in Figure 12.

As shown in Figure 12, at a central frequency of 900 MHz, the AMC-loaded metasurface achieves a gain improvement of 13.91 dB to 11.24 dB on the E-plane, with a maximum gain increase of 2.67 dB. The H-plane gain also improves from 2.34 dB to 2.69 dB, representing a 0.35 dB enhancement. The H-plane demonstrates superior performance after AMC loading, exhibiting more stable omnidirectional characteristics.

### 3.4. Physical Test

**Figure 13:**
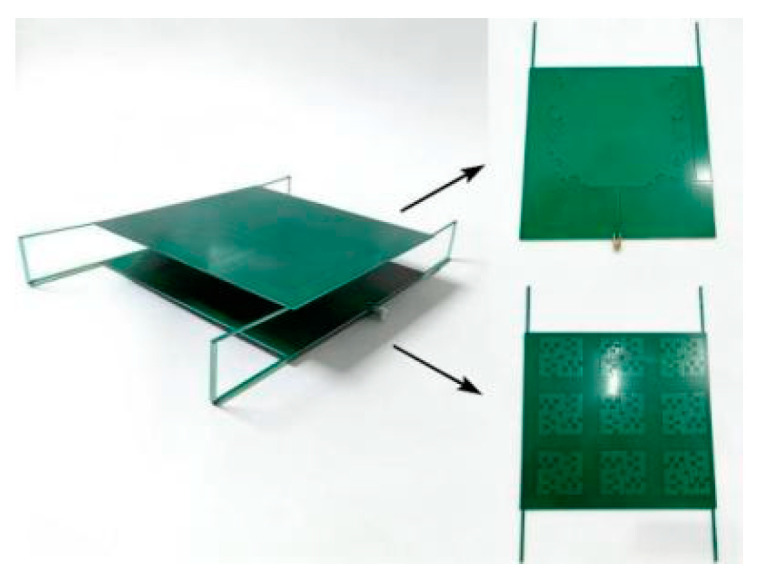
Physical diagram of the Koch fractal antenna.

The physical prototype of the Koch fractal antenna loaded with the AMC metasurface is illustrated in Figure 13, with the Koch fractal antenna on the upper layer and the loaded AMC metasurface on the lower layer.

**Figure 14:**
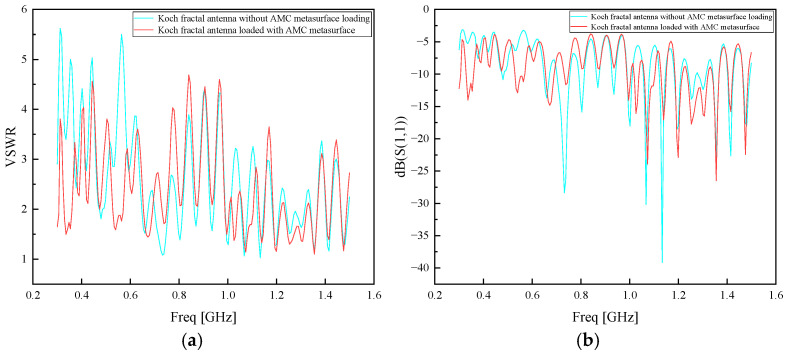
(**a**) Measured standing wave ratio curve of Koch fractal antenna. (**b**) Koch measurement of S11 parameters of fractal antenna.

After the physical prototype was completed, the antenna’s voltage standing wave ratio (VSWR) was tested using the Agilent E5063A vector network analyzer, with a frequency sweep range of 300 MHz to 1.5 GHz. The measured VSWR curves of the Koch fractal antenna and the Koch fractal antenna loaded with the AMC metasurface are shown in Figure 14a. The S11 curve is shown in Figure 14b. The standing wave ratio of the Koch fractal antenna is around 5 in the low-frequency band and below 5 in the mid- and high-frequency bands. In the S11 parameter chart, it is below −3.52 dB across all bands. After loading the AMC metasurface, the standing wave ratio remains below 5 across the full frequency range, and in the S11 parameter chart, it remains below −3.52 dB.

**Figure 15:**
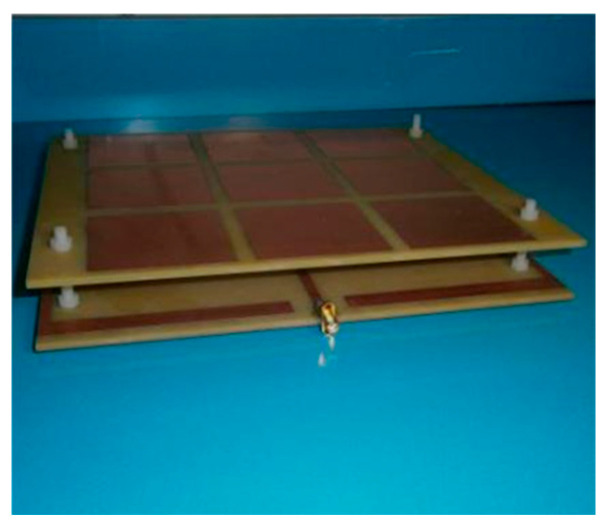
Resonant cavity monopole UHF antenna sensor.

This study employs the resonant cavity monopole UHF antenna sensor designed in Reference [27] (as shown in Figure 15) as the reference antenna for experimental comparison. The VSWR of this antenna is less than 5 across the frequency ranges of 0.41 GHz to 1.69 GHz and 1.75 GHz to 3 GHz. The antenna achieves a maximum gain of 5.54 dB at 0.7 GHz, 7.16 dB at 1.0 GHz, and 3.46 dB at 1.4 GHz.

**Figure 16:**
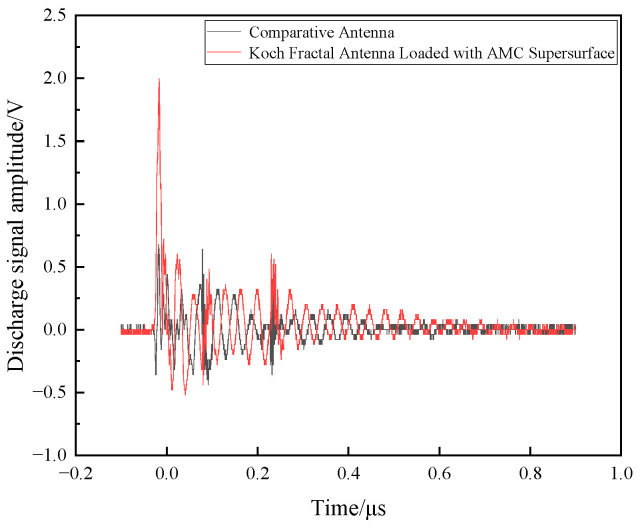
Comparison of discharge signal amplitude between antenna and Koch fractal antenna loaded with AMC metasurface.

In the electrostatic discharge experiment, the experimental circuit diagram is shown in Figure 10 of our group’s previously published literature [40]. The reference antenna, Koch fractal antenna, and Koch fractal antenna with AMC metasurface were spaced 30 cm apart, with the electrostatic discharge gun positioned at the center of the three antennas. Figure 16 shows the comparison between the measured discharge signal amplitude of the reference antenna and that of the Koch fractal antenna loaded with the AMC metasurface.

As shown in Figure 16, the maximum amplitude of the discharge signal measured by the reference antenna was 680 mV, while the maximum amplitude measured by the Koch fractal antenna equipped with AMC metasurface reached 2 V. Both antennas clearly identified the discharge signal, but the Koch fractal antenna demonstrated higher amplitude and greater detection sensitivity.

**Figure 17:**
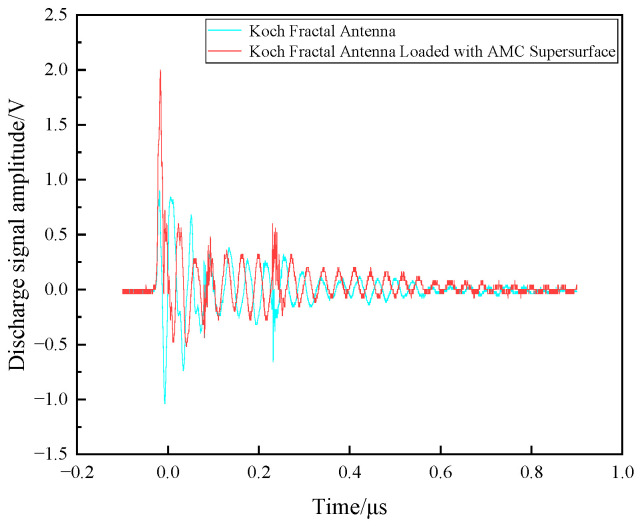
Comparison of discharge signal amplitude measured before and after loading the AMC metasurface on the Koch fractal antenna.

Figure 17 shows a comparison between the discharge signal amplitude measured by the Koch fractal antenna and that measured after loading the AMC metasurface.

As shown in Figure 17, the maximum amplitude of the discharge signal measured by the Koch fractal antenna without the AMC metasurface was 900 mV, while the maximum amplitude measured by the Koch fractal antenna with the AMC metasurface was 2 V. It can be observed that the Koch fractal antenna with the AMC metasurface exhibits greater sensitivity in detecting discharge signals, with a maximum amplitude reaching 122.2% of that measured by the antenna alone.

Ten sets of electrostatic discharge signal amplitude data were measured for the antenna compared with the Koch fractal antenna loaded with AMC metasurface, and for the Koch fractal antenna without AMC metasurface compared with the Koch fractal antenna loaded with AMC metasurface. The results obtained by averaging these ten sets of data are shown in Table 5.

To further validate the practical performance of Koch fractal antennas in detecting UHF partial discharge signals, a laboratory test platform simulating typical GIS partial discharge defects was established. Comparative tests were conducted between Koch fractal antennas loaded with AMC metasurfaces and reference antennas, as well as between Koch fractal antennas and Koch fractal antennas loaded with AMC metasurfaces.

The schematic diagram of the laboratory-built GIS typical defect partial discharge simulation test circuit is shown in Figure 13 of our group’s previously published literature [10]. This test circuit consists of a power frequency AC power supply, filter, test transformer control console, power frequency test transformer, protective resistor, voltage divider, and a cavity simulating the actual GIS structure. The test transformer, protective resistor, and voltage divider circuit are all partial discharge-free equipment. The UHF detection antenna is positioned at the observation port of the GIS simulation chamber tank. During testing, a needle–plate partial discharge model is installed within the GIS simulation chamber to simulate internal metallic protrusions. The chamber is filled with SF6 gas at 0.4 MPa. Signal acquisition utilizes a Tektronix* MS044 (Beaverton, OR, USA) high-performance digital storage oscilloscope with four channels.

**Figure 18:**
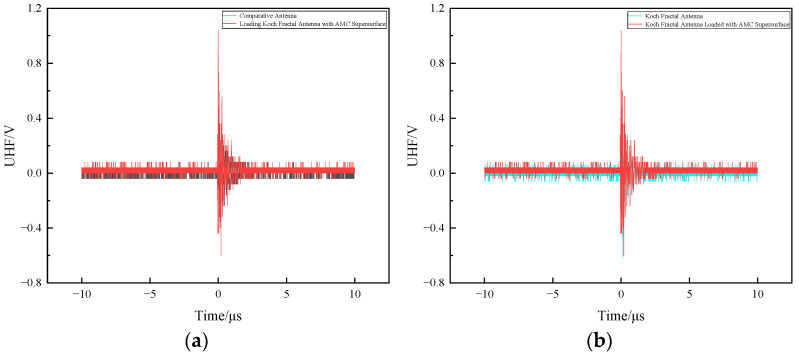
(**a**) Comparison of UHF signal amplitude measured between conventional antennas and Koch fractal antennas loaded with AMC metasurfaces. (**b**) Comparison of UHF signal amplitude measurements between Koch fractal antennas and Koch fractal antennas loaded with AMC metasurfaces.

First, the reference antenna, Koch fractal antenna, and Koch fractal antenna loaded with AMC metasurface were all placed at the observation port of the simulation cavity tank. At a voltage of 2.45 kV, the measured UHF signal amplitude of the reference antenna was compared with that of the Koch fractal antenna loaded with the AMC metasurface, as shown in Figure 18a. Figure 18b shows the comparison between the UHF signal amplitude measured by the Koch fractal antenna and that measured by the Koch fractal antenna loaded with the AMC metasurface. The reference antenna recorded a maximum UHF signal amplitude of 360 mV, while the Koch fractal antenna recorded a maximum amplitude of 420 mV. The Koch fractal antenna with the AMC metasurface achieved a maximum UHF signal amplitude of 1.040 V. This demonstrates that the Koch fractal antenna with the AMC metasurface exhibits higher sensitivity.

In the GIS insulation defect discharge experiment, the reference antenna and Koch fractal antenna were compared against the Koch fractal antenna loaded with an AMC metasurface. Ten sets of UHF signal amplitude data were measured at different voltage values. The results obtained by averaging these ten sets of data are shown in Table 6.

## 4. Conclusions and Discussion

To address the low sensitivity issue of external UHF antennas, this paper proposes a Koch complementary fractal UHF antenna based on AMC metasurfaces. A Koch fractal antenna gain-enhancing AMC metasurface was designed, and a multi-parameter joint optimization method was employed to obtain the optimal structural parameter set for the Koch fractal antenna loaded with the AMC metasurface. Finally, after experimental comparison and analysis of the Koch complementary fractal UHF antenna, the following conclusions were drawn:

1. The standing wave ratio of the Koch fractal antenna decreased after loading the AMC metasurface, achieving better impedance matching. The mean gain increased by approximately 1.52 dB, representing about 35.19% of the original antenna’s gain. The maximum gain improvement reached approximately 5.2 dB, equivalent to about 120% of the original antenna’s maximum gain. In electrostatic gun discharge experiments, the Koch fractal antenna loaded with AMC metasurfaces demonstrated a 91.32% higher UHF signal amplitude detection capability compared to the original Koch fractal antenna.
2. In insulation defect discharge experiments, Koch fractal antennas loaded with AMC metasurfaces detected UHF signal amplitudes at 2.45 kV, 4.10 kV, 6.20 kV, 8.40 kV, and 10.18 kV. Therefore, the Koch complementary fractal antenna equipped with the AMC metasurface achieves greater UHF signal amplitude detection, higher sensitivity, and superior performance.

## Figures and Tables

**Table 1:** Dimensions of the Koch fractal antenna.

Parameter	Value/mm	Parameter	Value/mm
*a*	180	*W*_1_	11.8
*b*	180	*W*_2_	120
*L*	58.3	*W*_3_	1.07
*L*_1_	150	*W*_4_	47.6
*L*_2_	15.372	*h*_0_	1.6
*L*_3_	50	*h*_1_	12
*L*_4_	15.75	/	/

**Table 2:** Dimensions of the AMC metasurface unit.

Parameter	Value/mm	Parameter	Value/mm
*L*_5_	184	*W*_6_	0.9
*L*_6_	53	*W*_7_	11.7
*L*_7_	11.1	*W*_8_	5.6
*L*_8_	2	*h*_2_	1
*L*_9_	2.5	*h*_3_	30
*L*_10_	11	*e*	0.6
*W*_5_	184	/	/

**Table 3:** Optimization of dimensional parameters for AMC metasurface unit cells.

Parameter	Original Values/mm	Optimization Range/mm	Step Size/mm
*L*_7_	11.10	8.10~11.1	1.0
*W*_7_	11.70	10.7~12.7	1.0
*e*	0.600	0.50~0.70	0.1
*L*_9_	2.500	2.40~2.60	0.1
*W*_6_	0.900	0.80~1.00	0.1
*W*_8_	5.600	5.50~5.70	0.1
*L*_8_	2.000	1.90~2.10	0.1
*L*_10_	11.00	10.9~11.1	0.1
*h*_3_	30.00	20.9~30.1	0.1
*L*_11_	20.00	40.0~60.0	10

**Table 4:** Parameter values prior to and subsequent to optimization.

Parameter	Before Optimization/mm	After Optimization/mm
*L*_7_	11.10	9.40
*W*_7_	11.70	11.80
*e*	0.600	0.600
*L*_9_	2.500	2.500
*W*_6_	0.900	0.900
*W*_8_	5.600	5.600
*L*_8_	2.000	2.000
*L*_10_	11.00	11.00
*h*_3_	30.00	30.00
*L*_11_	20.00	50.00

**Table 5:** Detect the average amplitude of the electrostatic gun discharge signal.

Comparative Antenna/V	Koch Fractal Antenna/V	Loading Koch Fractal Antenna with AMC Supersurface/V	Amplitude Increase
0.628	0.922	1.764	91.32%

**Table 6:** Average amplitude of UHF discharge signals from GIS insulation defects.

Voltage/kV	Koch Fractal Antenna/V	Loading Koch Fractal Antenna with AMC Supersurface/V	Amplitude Increase
2.45	0.432	0.968	124.07%
4.10	0.376	1.004	167.02%
6.20	0.408	1.012	148.04%
8.40	0.428	1.040	142.99%
10.18	0.448	1.080	141.07%

**Figure A1:**
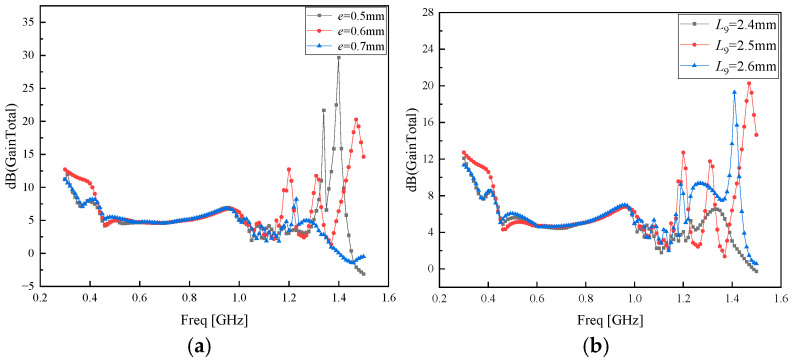

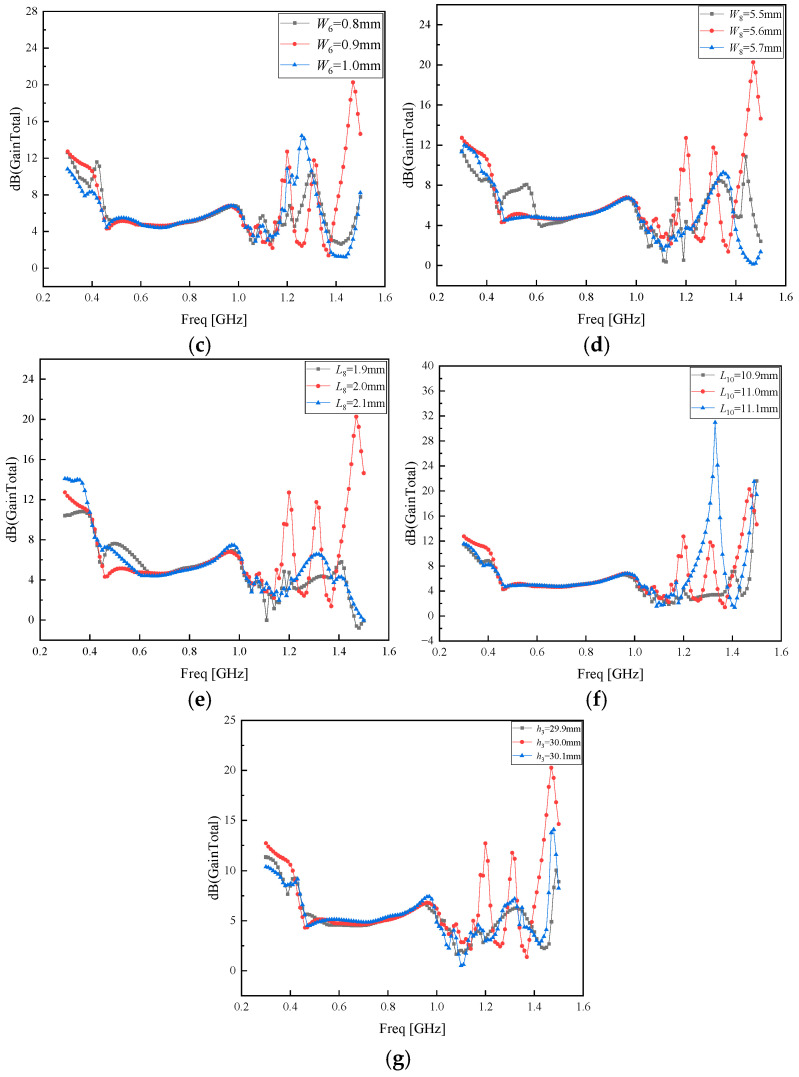
(**a**) Optimized parameter e. (**b**) Optimized parameter L9. (**c**) Optimized parameter W6. (**d**) Optimized parameter W8. (**e**) Optimized parameter L8. (**f**) Optimized parameter L10. (**g**) Optimized parameter *h*_3_.

## Data Availability

The data are available upon request from the corresponding author.

## Appendix A

The optimization of the remaining parameters of the AMC super-surface is shown in Figure A1.
